# Evaluation and review of body fluids saliva, sweat and tear compared to biochemical hydration assessment markers within blood and urine

**DOI:** 10.1038/ejcn.2017.136

**Published:** 2017-08-30

**Authors:** M Villiger, R Stoop, T Vetsch, E Hohenauer, M Pini, P Clarys, F Pereira, R Clijsen

**Affiliations:** 1 Department of Business Economics, Health and Social Care, University of Applied Sciences and Arts of Southern Switzerland, Landquart/Manno, Switzerland; 2 THIM University of Applied Sciences, Landquart, Switzerland; 3 Department of Movement and Sport Sciences, Vrije Universiteit Brussel, Brussels, Belgium; 4 Faculty of Medicine, Imperial College London, London, Great Britain; 5 CSEM Centre Suisse d’Electronique et de Microtechnique SA, Landquart, Switzerland

## Abstract

Evaluating and testing hydration status is increasingly requested by rehabilitation, sport, military and performance-related activities. Besides commonly used biochemical hydration assessment markers within blood and urine, which have their advantages and limitations in collection and evaluating hydration status, there are other potential markers present within saliva, sweat or tear. This literature review focuses on body fluids saliva, sweat and tear compared to blood and urine regarding practicality and hydration status influenced by fluid restriction and/or physical activity. The selected articles included healthy subjects, biochemical hydration assessment markers and a well-described (de)hydration procedure. The included studies (*n*=16) revealed that the setting and the method of collecting respectively accessing body fluids are particularly important aspects to choose the optimal hydration marker. To obtain a sample of saliva is one of the simplest ways to collect body fluids. During exercise and heat exposures, saliva composition might be an effective index but seems to be highly variable. The collection of sweat is a more extensive and time-consuming technique making it more difficult to evaluate dehydration and to make a statement about the hydration status at a particular time. The collection procedure of tear fluid is easy to access and causes very little discomfort to the subject. Tear osmolarity increases with dehydration in parallel to alterations in plasma osmolality and urine-specific gravity. But at the individual level, its sensitivity has to be further determined.

## Introduction

Human health and performance can be reduced when the body is dehydrated^1, 2^ and no gold standard for hydration assessment exists.^3, 4^ Dehydration is a result of excess total body water (TBW) loss and is often accompanied by abnormalities in electrolyte balance. Heat, exercise-induced sweating or reduced thirst (often found in the elderly population) cause more water than sodium loss from the extracellular fluid (ECF) compartment, that is, hypertonic dehydration. Hypotonic dehydration shows more sodium than water loss and can be induced by diuretics or severely burned skin. Isotonic dehydration is caused by water and sodium loss in equivalent proportions such as during diarrhoea.^5^ When people exercise for longer periods without fluid replacement, whole-body dehydration is associated with reductions in plasma, interstitial and intracellular volume, that is, hypovolemia.^6^

Changes in body mass (BMc) are often used as measures of (de)hydration.^7^ This measurement is simple, inexpensive and noninvasive. Poorer memory and attention is observed with a BMc of <1%,^8^ diminished physiological performance with a BMc of >1–2%^9, 10, 11, 12, 13, 14^ and impaired endurance exercise performance with a dehydration threshold of ⩾2%.^15^ Measured fasting morning body mass is a reliable index of euhydration when assessed during 3–9 consecutive days,^16, 17^ but the measurement of BMc has also some limitations. Measurements across the required 3–9 days are not always feasible (for example, availability) and for people participating in an exercise programme, body mass can change over time as a consequence of muscle hypertrophy independently of changes in body water or body fat loss.^18^ Thus, where it is not practical or possible to use repetitive measurements of body mass over time (monitoring), biochemical indices of hydration status based on concentration and composition of body fluids (that is, blood and urine) become a necessary alternative to evaluate body fluid balance.^19^

Blood plasma osmolality (BP_osm_—concentration of plasma, milliosmoles of solute particles per kilogram of water), plasma sodium concentration BP[Na^+^] or blood serum osmolality (BS_osm_) are regularly used blood markers for evaluating hydration status.^20, 21, 22, 23^ In this regard, when body fluids are steady and balanced, BP_osm_ combined with the TBW (fluid in intracellular and extracellular areas; approximately 0.6 l/kg≈63.3% of body mass) value describe the most accurate and exact hydration assessment values.^3^ But these hydration assessment techniques, such as stable isotope dilution and neutron activation analysis for TBW and BP_osm_, are time-consuming, expensive and invasive. Fluid concentrations are regulated in complex and dynamic processes. The intracellular fluid is measured indirectly by means of the assumption that the isotope distributes equally throughout intracellular fluid and ECF^3^ and that the hydration status is changed by volume and timing of water, sodium and osmolyte consumption.^24^

As blood sampling is often difficult to execute in the field, evaluating hydration status in these settings include determination of urine osmolality (UR_osm_), urine-specific gravity (UR_sg_) and urine colour (UR_col_).^25, 26^ UR_sg_ seems to be the most valid marker in the setting of dynamic (monitoring over time) dehydration assessment among these urinary markers.^20^

To be of practical use, measuring and evaluating hydration status should have the possibility to be used on a daily or even hourly basis. The ability to monitor hydration status has become increasingly studied within the rehabilitation, sport, military and performance-related activities.^27, 28, 29, 30^ Besides BMc, BP_osm_ and UR_sg_ seem to be the most effective markers to monitor hydration status among the aforementioned markers.^20^

There might be other potential biochemical markers present within saliva, sweat and tears being noninvasive ‘freely accessible’ body fluids.^31, 32^ These markers can offer further in-depth knowledge of an individual’s hydration status. On this basis, the interest of this review is to evaluate ‘freely accessible’ body fluids (saliva, sweat and tear) as hydration assessment markers compared to the aforementioned body fluids (blood: BP_osm_, BP[Na^+^], BS_osm_; urine: UR_osm_, UR_sg_, UR_col_) during a well-described (de)hydration procedure influenced by fluid restriction and/or physical activity.

## Materials and methods

### Search strategy and selection process

A systematic search was accomplished in 2016 in the electronic databases MEDLINE (PubMed) and CENTRAL (Cochrane Library).

The following keywords and combinations using the Boolean formula AND/OR were used in PubMed without applying any automatic filters: (hydration OR dehydration [MeSH] OR hypernatremia [MeSH] OR hyponatremia [MeSH] OR water loss [MeSH] OR body fluid balance [MeSH]) AND (saliva [MeSH] OR sweat [MeSH] OR tear [MeSH] OR axillary moisture OR skin temperature [MeSH] OR skin humidity [MeSH] OR skin water loss [MeSH] OR skin hydration) AND (body composition [MeSH] OR body weight [MeSH] OR body mass OR urine [MeSH] OR blood [MeSH]).

The following keywords and combinations were used in Cochrane Library (CENTRAL): dehydration AND saliva, dehydration AND sweat, dehydration AND tear, dehydration AND body weight and dehydration AND axillary moisture.

Records were excluded according to the below-mentioned inclusion/exclusion criteria. After removing all duplicates, titles and abstracts were checked by MV, RS, TV, EH, MP and RC. In addition, full texts of rejected records were randomly checked by the testers to ensure validity of the inclusion/exclusion criteria. Inclusion criteria for the articles were as follows: (1) randomized clinical or clinical trials, (2) English or German, (3) healthy humans regardless of age or physical performance ability, (4) biochemical hydration assessment markers (blood: BP_osm_, BP[Na^+^], BS_osm_ and/or urine: UR_osm_, UR_sg_, UR_col_), (5) BMc and (6) ‘freely accessible’ and direct evaluation of body fluids (saliva, sweat, tear and axillary moisture) used as hydration assessment markers. In addition, the studies were checked for a well-described dehydration procedure (influenced by fluid restriction and/or physical activity).

Besides the search in the electronic databases, records were checked through links of related articles and references.

### Measurement equipment and quantity estimates

For evaluating hydration status, osmolarity (mOsmol/l) and osmolality (mOsmol/kg) are mainly measured by freezing-point depression.^20^ The freezing points of solutions are lower with regard to a pure solvent and is directly proportional to the molality of the solute. Under the control of an osmometer, controller samples are allowed to melt relatively slowly and the freezing point can be determined: BP_osm_: 275–295 mOsmol/kg (euhydrated),^20, 32, 33, 34^ BS_osm_: 282–295 mOsmol/kg (euhydrated),^35^ and UR_osm_: <700 mOsmol/kg (euhydrated).^13, 36^

An ion-selective electrode measures the potential of a specific ion in solution (mmol/l): BP[Na^+^]: 135–145 mmol/l (euhydrated).^22, 23^

Specific gravity (g/ml) is the ratio of the density of a substance to the density of a reference substance and is measured by a refractometer: UR_sg_: <1.010 g/ml (euhydrated).^13, 14^

The urine colour is measured by an ‚8-point colour chart’: UR_col_: 1 or 2 (euhydrated).^14^

## Results

**Figure 1:**
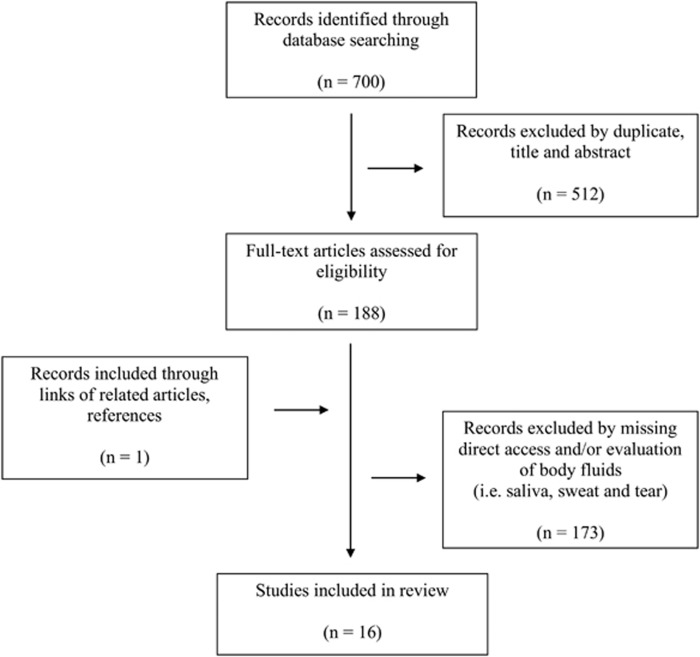
Flowchart of study screening and selection.

In the Cochrane Library *n*=388 studies were recorded: dehydration AND body weight (279 hits), dehydration AND saliva (19 hits), dehydration AND sweat (75 hits), dehydration AND tear (15 hits), and dehydration AND axillary moisture (0 hits). In PubMed *n*=312 studies were found. After excluding records by duplicates and by not relevant titles, abstracts and full texts, 15 records met our inclusion criteria and one record was included through links of related articles (references). In summary, a total of *n*=16 studies was included in this review. Figure 1 presents the search strategy and selection process.

### Dehydration procedures

In this section the dehydration procedures of the included studies of saliva (Table 1), sweat (Table 2) and tear are listed. To achieve an euhydrated state before testing all studies conducted a well-described hydration protocol. For a controlled dehydrated status, either a stationary cycle ergometer was used in an environmental chamber (saliva,^20, 28, 36, 37, 38^ sweat^19, 39, 40^ and tear^27, 31^) or a treadmill (saliva^20, 28, 29, 34, 41^ and sweat^34^). Passive dehydration was either used with fluid restriction^42, 43^ or extracellular dehydration using a loop diuretic (Furosemide).^44^ For comparison reasons, the results stand for the control/placebo groups to evaluate (de)hydration status.

### Measurement equipment and direct fluid collection of saliva, sweat and tear

For most of the included studies, the measurement equipment to evaluate osmolarity and sodium concentration of saliva and sweat do not differ compared to the evaluation of blood and urine.^19, 20, 28, 29, 34, 36, 37, 39, 40, 42, 44^

Regarding saliva, Smith *et al.*^41^ (SA_osm_ and UR_osm_: Fiske One-Ten Osmometer, Norwood, MA, USA; BP_osm_: Model 3320 Osmometer) and Taylor *et al.*^38^ (SA_osm_: Fiske One-Ten Osmometer, Norwood, MA, USA; BS_osm_ and UR_osm_: Model 3250 Osmometer) used not the same osmometer to evaluate SA_osm_, BP_osm_ and UR_osm_.

#### Saliva fluid

SA_osm_ was measured after centrifugation by a freezing-point depression osmometer (for example, Fiske Associates, Fiske Micro-Osmometer, Model 210/Fiske One-Ten Osmometer^20, 28, 38, 41, 44^/Advanced Instruments, Model 3320/Model 3MO, Norwood, MA, USA^29, 36, 37, 42^) and SA[Na+] was measured by an ion-selective electrode (Beckman Synchron EI-ISE, Fullerton, CA, USA^34^). Direct collection of saliva samples is possible in two ways as described in the study by Ely *et al.*^44^

Saliva samples (expectorated^20, 28, 44^): Particiapnts were initially asked to swallow followed by a period of 2 min of passive saliva collection. The collected saliva was then expelled (spit) into a polypropylene Falcon tube (for example, Voigt Global Distribution, Inc., Lawrence, KS, USA).

Saliva samples (salivette^29, 43, 44^): Participants were asked to swallow before saliva collection. Saliva was collected with the use of a pre-weighed polyester salivette swab (for example, Sarstedt, Leics, UK) which was placed under the tongue for 2 min. During the collection period participants avoided any orofacial movements. To obtain salivary concentration samples, the participants were asked to accumulate saliva in their mouth (that is, passive drool technique) and finally expel around 1 ml of saliva into a Dixie cup.

*Sweat* can be directly collected via two different techniques: (1) absorbancy method (i.e. sweat patch collection); and (2) whole-body washdown technique.^34^

(1) For the absorbancy method, the patch location was first cleaned (alcohol and distilled water) and then dried (air). Afterwards sweat patches were used where they were needed (for example, side-by-side to the upper back, just below the shoulder blades, to the forearm, chest and mid-thigh on the right-hand side—the patches remained in place throughout the trial).^34, 40^ Sweat was measured in small batches by for example a Cobas C311 module (Roche Diagnostics, Basel, Switzerland) using the ion-selective electrode technique for SW[Na^+^] (Easylyte Plus, Medica Corporation, Bedford, MA, USA,^19^ Beckman Synchron EI-ISE, Fullerton, CA, USA,^34^ Beckman Instruments Inc., AS 80 System, Galway, Ireland^39^) or flame photometry (Sherwood, Cambridge, UK^40^).

(2) For the whole-body washdown technique, a kiddie pool placed in a fully enclosed walk-in tent was prepared in advance. After completion of the performance trial, each participant entered the plastic kiddie pool and approximately 1.5 l of distilled water was poured over the participant’s head and body.^34^ The participant was then asked to remove all clothing in the privacy of the tent and then pour the remaining amount of distilled water (3.78 l total) over his or her body. The removed clothes remained in the kiddy pool and to obtain water samples Eppendorf tubes were used.

*Tear fluid* was collected and analysed for TE_osm_ using a commercially available diagnostic device (TearLab Osmolarity System; TearLab, San Diego, CA, USA).^27, 31^ Participants blinked three times and squeezed their eyes shut. The released tear fluid from the lacrimal gland was immediately collected from the right eye using a handheld pen. A signal was transmitted when a sufficient volume (50 nl) was collected, which typically took <5 s. Once docked on the TearLab platform, the outcome was presented within 10 s. BP_osm_ was measured by a freezing-point depression osmometer (Model 2020^27^/Model 330 MO,^31^ Advanced Instruments).

## Discussion

The aim of the review was to evaluate ‘freely accessible’ and noninvasive body fluids (saliva, sweat and tear) compared to biochemical hydration assessment markers such as those within blood and urine during a well-described (de)hydration procedure influenced by fluid restriction and/or physical activity. First, the practical use of the different hydration assessment markers and, second, the results of saliva, sweat and tear body fluids are discussed regarding hydration status.

### Practical use of body fluid hydration assessment markers

The measurement equipment for the evaluation of the hydration status change did not differ substantially between the included studies. This means that the underlying methods to evaluate osmolarity, osmomality^20^ and sodium concentration^22^ of body fluids were comparable. In this regard, not only the assessment technique itself but the procedure of collecting body fluids has a fundamental impact on the use of the biochemical hydration assessment markers. Collecting blood is an invasive procedure that makes it often difficult to execute in the field. Furthermore, BP_osm_ and BS_osm_ are tightly regulated in the brain. They are not good indices of the hydration status across days but across hours, because the kidneys constantly attempt to bring tonicity back below 296 mOsmol/kg.^45^

Urine collection is a noninvasive method. A limiting factor during the dehydration process is the availabilty of urine (for example, bladder voiding is not always feasible). Measuring UR_sg_ with a refractometer is less subjective than UR_col_ as well as simple to use.^14^ Although UR_osm_, UR_sg_ and UR_col_ have been suggested for screening older adults for dehydration, their diagnostic accuracy is too marginal to be beneficial.^46^ In addition, a dehydration procedure for older adults is not reasonable. Compared to blood and urine, saliva samples can always be directly collected and are always available. But saliva markers seem to be highly variable between subjects (see below). Futhermore, there are two possible techniques to collect sweat—the absorbancy method (that is, sweat patch collection) or the whole-body washdown technique.^34^ Compared to the collection of the other body fluids both techniques provide no baseline measurement. The collection of sweat is time-consuming and the sweating protocol only practicable in sweating-related activities rather than rehabilitation or nursing setting. Although only two studies with the collection procedure of tear fluid were included in this review, the procedure shows a promising result. The collection of tear fluid causes little discomfort to the subject and a sample is always obtained.^27, 31^

The interested reader with regard to cost efficiency, time efficiency, simplicity of test and scientific value of hydration assessment markers is directed elsewhere.^47^

### Saliva and hydration status

Saliva is made up mostly of water (97–99.5%) originating from plasma via acinar cells.^48^ The accumulation of primary saliva is supported by the transacinar cell sodium gradient from plasma through acinar cells. The ECF sodium concentration increases and this is reflected in an increase in BP_osm_ during hypertonic-hypovolemia dehydration what might be linked with the secretion of more concentrated saliva with a decrease in TBW.^36^ SA_osm_ has been shown to increase with progressive dehydration,^28, 36, 38^ fluid deprivation and restriction.^29, 43^ Furthermore, to record alterations during hypertonic-hypovolemia dehydration, SA_osm_ might be as sensitive as UR_osm_. Given that a fluid intake of 1.0 l per day seems to be insufficient to compensate water losses during the day,^49^ it was assumed that there would be differences between the low and high fluid intake regarding SA_osm_.^42^ However, no differences in SA_osm_ were reported by Perrier *et al.*^42^ In this regard SA_osm_ was highly variable between participants as also shown in prior studies.^28, 36^

During active heat exposure in the study by Muñoz *et al.*,^37^ BS_osm_ and SA_osm_ were the most effective hydration assessment markers (that is, high specificity and sensitivity). Further, for single measurements, BS_osm_ and SA_osm_ propose good usability during high temperature and exercise. For measurements over time BS_osm_, UR_sg_ and BMc seem to be the most valid hydration assessment markers. In this regard, Cheuvront *et al.*^20^ suggest that BP_osm_, UR_sg_ and BMc are appropriate markers during dynamic (monitoring over time) dehydration but only BP_osm_ (not SA_osm_) as useful marker for static (one time) dehydration assessment.

There were weak significant correlations reported between SA[Na^+^] and BP[Na^+^] (*r*=0.45). Thus, the use of saliva provides limited support as a potential substitute for reporting changes in BP[Na^+^] in real time^36^ during exercise,^37^ probably because of reduced parasympathetic stimuli that alter secretion rates of saliva.^37^ However, the difference between BP[Na^+^] and SA[Na^+^] was approximately sevenfold.

### Sweat and hydration status

In a hot environment or during exercise, body temperature is controlled by the evaporation of sweat. The deficit in electrolytes can be preserved by means of sodium reabsorption from the duct of sweat glands. Evidence supporting blood osmolality as a hydration assessment marker usually comes from studies that integrate a sweat-loss model of hypertonic-hypovolemia in young, fit and healthy individuals. In this regard, blood osmolality is unsuitable to detect isotonic-hypovolemia often following from illness and medications (for example, diuretics) in a clinical setting.^47^

Hew-Butler *et al.*^34^ utilized both abovementioned techniques to quantify changes in SW[Na^+^]. But the lack to detect small, real-time, regulatory variations in sweat gland output limits the study. However, SW[Na^+^] values were sevenfold less than the patch collection (between 12 and 14 mmol/l) and ~50% less than those previously indicated. The differences between the absorbancy method and the whole-body washdown technique results may be due to missed collection of sweat, captured electrolytes in clothes and/or collection area, or contamination from other solutions.

A further limitation of the collection of sweat is the missing baseline value. To compare a dehydrated with an euhydrated status, Morgan *et al.*^40^ tested participants ingesting either no fluid (dehydration) or a 20 mmol/l sodium chloride solution (euhydration) during exercise. They showed that dehydration caused an increase in SW[Na^+^] with regard to an euhydrated state. It is proposed that differences between dehydration and euhydration in ECF[Na^+^], acute aldosterone and sympathetic nervous activity could cause the changed sweat composition. Both BS[Na^+^] and SW[Na^+^] were higher in a dehydrated state.^40^ When the subjects were dehydrated due to higher BS[Na^+^], it is predicted by Morgan *et al.*^40^ that an increased sodium concentration would have appeared in the primary sweat. However, the difference between dehydration and euhydration for BS[Na^+^] was approximately 3 mmol/l, which was lower than that found in sweat, which was ~10 mmol/l. Therefore, a simple displacement into primary sweat, resulting from the greater ECF[Na^+^] could not solely account for the higher SW[Na^+^]. A second possible explanation for the increase in SW[Na^+^] during dehydration could have been an influence of elevated aldosterone on the secretory coil. A third explanation for the increase in SW[Na^+^] could also have been an augmented activity of the sympathetic nervous system. During exercise-induced dehydration, the calculated ECF volume by Hamouti *et al.*^19^ declined progressively from exercise-baseline value likely due to water losses through sweating while BS_osm_ increased. Furthermore, SW[Na^+^] losses, as a result of a higher SW[Na^+^] concentration, can significantly affect post-race BS[Na^+^] concentration in long-runners,^50^ and SW[Na^+^] did not reflect the same pattern as UR[Na^+^].^34^

### Tear and hydration status

Tear fluid is a complex solution intended to sustain the surface of the eye.^51^ The lacrimal gland secretes tear fluid consisted mainly of water and electrolytes, and human tears have been disclosed to be isotonic with plasma.^52^

TE_osm_ increased with dehydration and tracked changes in BP_osm_ and UR_sg_, and therefore it might offer a new hydration assessment technique in rehabilitation, sport, military and performance-related activities. TE_osm_ can record alterations in hydration status due to water consumption during progressive rehydration following exercise as well as differentiate between dehydration (2–3% BMc) and euhydration during exercise.^27^ It seems that BP_osm_ and TE_osm_ have the strongest correlation over the other widely used hydration assessments (eg. BMc, UR_sg_).

It is suggested that a TE_osm_ value >309 mOsmol/l reflects dehydration.^31^ This value was not reached in the two included studies but increases in BP_osm_ during exercise-evoked dehydration^27, 31^ and subsequent overnight fluid restriction^31^ were represented in increases in TE_osm_. The data indicate that TE_osm_ was ~5–10 mOsmol higher than the respective BP_osm_ (Table 3). The BP_osm_ cutoff for minimal dehydration is 295 mOsmol/kg.^14^ Compared with the TE_osm_ values, 301 mOsmol/l could be the cutoff value for a minimally dehydrated status. It has to be taken into account that TE_osm_ was measured as osmolarity’ (number of osmoles of solute per liter of solution) and BP_osm_ was measured as osmolality’ (number of osmoles of solute per kilogram of solvent). Furthermore, in the response of TE_osm_ to changes in hydration status, there are large differences among subjects limiting its validity and usefulness at the individual level. The potential usefulness of TE_osm_ to estimate hydration status at the individual level has to be further determined as well as how its validity and reliability are impacted by field conditions.^27^ Nevertheless, it can be suggested that TE_osm_ has utility as a marker of hydration status (strong correlation between TE_osm_ and BP_osm_: *r*=0.93). The correlation between TE_osm_ and BP_osm_ was even stronger than that between U_sg_ and BP_osm_ (r=0.72).^31^

### Dehydration and hyperthermia

Trangmar and González-Alonso^6^ showed that progressive exercise-induced dehydration, with concomitant hyperthermia, can be associated with impaired perfusion to tissues and organs. In most included studies, the combination of exercise-induced dehydration and heat stress was presented, which makes it difficult to separate the effects of dehydration and hyperthermia in each compartment.^53^ It is well known that hyperthermia negatively influences endurance performance,^54^ but the effect on short-term high-intensity performance is still unclear.^55^ Thermoregulatory functions depend on sufficient body water. Consequently, losses in TBW can challenge the thermoregulatory system. A deficit of TBW with a BMc of ⩾2% (dehydration) is the threshold for measurably altered thermoregulation.^56^

Recent evidence further complicates the assessment of hydration status, in that different hydration assessment markers may validly identify dehydration in one circumstance but not another.^37, 47^

### Limitations

When interpreting data, one should be aware of the relative small number of studies. Although there are many studies about (de)hydration, some aspects differ substantially such as dehydration procedures and used hydration assessment markers. First, to achieve an euhydrated state before testing, all studies had to conduct a well-described hydration protocol and afterwards a well-described dehydration procedure (influenced by fluid restriction and/or physical activity). This limited the number of studies with tear fluid for example. Second, this review has focused on ‘freely accessible’ and direct evaluation of body fluids saliva, sweat and tear. Thus, saliva, sweat and tear could be directly compared to the other ‘standardized’ biochemical hydration assessment markers (see above) regarding osmolarity, osmomality^20^ and sodium concentration^22^ of body fluids. In particular, sweat as a hydration assessment marker was often indirectly evaluated.

## Conclusion

In summary, the setting and the method of collecting respectively accessing body fluids determine the use of a biochemical hydration assessment marker. Compared to other body fluids (for example, blood) obtaining a sample of saliva is one of the simplest ways to collect body fluids. During exercise and heat exposures, saliva might be an effective index to evaluate hydration status but seems to be highly variable and should be carefully used as a substitute marker of other biochemical hydration assessment markers. The lack of a baseline measurement and the time-consuming collection of sweat makes it more difficult to evaluate dehydration and to make a statement about the hydration status at a particular time. The collection procedure of tears shows little discomfort to the participants and is easy to access. TE_osm_ can evalute changes in hydration status and increase with dehydration and recorded changes in BP_osm_ with comparable utility to UR_sg_. But with only two included studies, it has to be further determined whether TE_osm_ is sensitive enough to evaluate dehydration at the individual level as its validity and reliability.

## Figures and Tables

**Table 1:** General characteristics of the included studies—saliva

*Study*	*Dehydration procedure*	*Monitoring time (body mass)*	*Urine*	*Abs*	*Rel*	*Blood*	*Abs*	*Rel*	*Saliva*	*Abs*	*Rel*
*Cheuvront et al.*^20^	Cycling/running (indoor) and fluid restriction	Baseline (78.2)	UR_osm_: 614 UR_sg_: 1.018 UR_col_: 3.8	—	—	BP_osm_: 292	—	—	SA_osm_: 71	—	—
		BMc 2.5%	UR_osm_: 1018 UR_sg_: 1.028 UR_col_: 6.5	404 0.01 2.7	65.8 1 71.1	BP_osm_: 301	9	3.1	SA_osm_: 86	15	21.1
*Ely et al.*^28^	Cycling/running (indoor) and fluid restriction	Baseline (83.8)	UR_sg_: 1.018	—	—	BP_osm_: 291	—	—	SA_osm_: 58	—	—
		BMc 4%	UR_sg_: 1.028	0.01	1	BP_osm_: 303	12	4.1	SA_osm_: 96	38	65.5
*Ely et al.*^44^	Pharmaceutical (furosemide) and fluid restriction	Baseline (79.1)	UR_sg_: 1.015	—	—	BP_osm_: 289	—	—	SA_osm_: 68	—	—
		BMc 3%	UR_sg_: 1.021	0.006	0.6	BP_osm_: 292	3	1	SA_osm_: 77	9	13.2
*Hew-Butler et al.*^34^	Running (indoor) and fluid restriction	Baseline	UR_osm_: 600 UR[Na^+^]: 110	—	—	BP_osm_: 289 BP[Na^+^]: 143	—	—	SA[Na^+^]: 18	—	—
		BMc 1.3%	UR_osm_: 640 UR[Na^+^]: 80	40 30	6.7 27.3	BP_osm_: 294 BP[Na^+^]: 143.5	5	1.7	SA[Na^+^]: 20	2	11.1
		BMc 1.7%	UR_osm_: 640 UR[Na^+^]: 60	40 50	6.7 45.5	BP_osm_: 298 BP[Na^+^]: 144.5	9	3.1	SA[Na^+^]: 28	8	55.6
Muñoz *et al.*^37^	Cycling (indoor) and fluid restriction	Baseline	UR_osm_: 439 UR_sg_: 1.012	—	—	BS_osm_: 296	—	—	SA_osm_: 64	—	—
		BMc 1%	UR_osm_: 661 UR_sg_: 1.018	222 0.006	50.6 0.6	BS_osm_: 301	5	1.7	SA_osm_: 90	26	40.6
Oliver *et al.*^29^	Walking (indoor) and fluid restriction	Baseline (74.7)	—	—	—	BP_osm_: 293	—	—	SA_osm_: 54	—	—
		BMc 3%	—	—	—	BP_osm_: 307	14	4.8	SA_osm_: 73	19	35.2
Perrier *et al.*^42^	Fluid restriction	Baseline	UR_osm_: 331 UR_sg_: 1.009 UR_col_: 2.5	—	—	BP_osm_: 287	—	—	SA_osm_: 70	—	—
		Post	UR_osm_: 748 UR_sg_: 1.019 UR_col_: 4.2	417 0.01 1.7	126 1 68	BP_osm_: 290	3	1	SA_osm_: 70	0	0
Pross *et al.*^42^	Fluid restriction	Baseline	UR_sg_°: 1.023 UR_col_°: 5.9	—	—	BP_osm_: 294	—	—	SA_osm_: 57	—	—
		Post	UR_sg_°: 1.027 UR_col_°: 5.5	0.004 0.4	0.4 6.8	BP_osm_: 302	8	2.7	SA_osm_: 69	12	21.1
Smith *et al.*^41^	Running (indoor) and fluid restriction	Baseline (81.7)	UR_osm_: 443	—	—	BP_osm_: 289	—	—	SA_osm_: 70	—	—
		BMc 0.9%	UR_osm_: 756 UR_col_: 4.75	313	70.7	BP_osm_: 290	1	0.3	SA_osm_: 84	14	20
*Taylor et al.*^38^	Cycling (indoor) and fluid restriction	Baseline, trial 1 (79)	UR_osm_: 187.3 UR_sg_: 1.004 UR_col_: 1.2	—	—	BS_osm_: 287	—	—	SA_osm_: 75	—	—
		BMc 1%	—	—	—	—	—	—	SA_osm_°: 105	30	40
		BMc 2%	—	—	—	—	—	—	SA_osm_°: 125	50	66.7
		BMc 3%	—	—	—	BS_osm_: 290	3	1	SA_osm_°: 145	70	93.3
		BMc 6%	—	—	—	BS_osm_: 299	12	4.2	SA_osm_°: 210	135	180
Walsh *et al.*^36^	Cycling (indoor) and fluid restriction	Baseline (73.9)	UR_osm_: 353	—	—	BP_osm_: 289	—	—	SA_osm_: 50	—	—
		BMc 1%	UR_osm_°: 400	47	13.3	BP_osm_°: 292	3	1	SA_osm_°: 56	6	12
		BMc 2%	UR_osm_°: 700	347	98.3	BP_osm_°: 293	4	1.4	SA_osm_°: 73	23	46
		BMc 3%	UR_osm_: 728	375	106	BP_osm_: 298	9	3.1	SA_osm_: 105	55	110

Abbreviations: Abs (absolute difference to baseline); BM_C_ (body mass change, percentage change); BP_osm_/BS_osm_ (blood plasma/serum osmolality, mOsmol/kg); BP[Na^+^]/BS[Na^+^] (blood plasma/serum sodium concentration, mmol/l); Rel (relative difference to baseline); SA_osm_ (saliva osmolality, mOsmol/kg, euhydrated: <83 mOsmol/kg); SA[Na^+^] (saliva sodium concentration, mmol/l); UR_osm_ (urine osmolality, mOsmol/kg), UR[Na^+^] (urine sodium concentration, mmol/l); UR_sg_ (urine-specific gravity, g/ml); UR_col_ (urine colour, units); values estimated out of figures° italic=athletes/soldiers.

**Table 2:** General characteristics of the included studies—sweat

*Study*	*Dehydration procedure*	*Monitoring time (body mass)*	*Urine*	*Abs*	*Rel*	*Blood*	*Abs*	*Rel*	*Sweat*
*Hamouti et al.*^19^	Cycling (indoor) and fluid restriction	Baseline	UR_osm_°: 570 UR_sg_°: 1.017 UR[Na^+^]: 79	—	—	BS_osm_°: 282 BS[Na^+^]: 140.4	—	—	—
		BMc 2%	UR_osm_°: 760 UR_sg_°: 1.023 UR[Na^+^]: 73.9	190 0.006 5.1	33.3 0.6 93.5	BS_osm_°: 292 BS[Na^+^]: 143.1	10 2.7	103.5 101.9	SW[Na^+^]: 65
*Hew-Butler et al.*^34^	Running (indoor) and fluid restriction	Baseline	UR_osm_: 600 UR[Na^+^]: 110	—	—	BP_osm_: 289 BP[Na^+^]: 143	—	—	—
		BMc 1.3%	UR_osm_: 640 UR[Na^+^]: 80	40 30	6.7 27.3	BP_osm_: 294 BP[Na^+^]: 143.5	5 0.5	1.7 100.3	SW[Na^+^]: 80
		BMc 1.7%	UR_osm_: 640 UR[Na^+^]: 60	40 50	6.7 45.5	BP_osm_: 298 BP[Na^+^]: 144.5	9 1.5	3.1 1.0	SW[Na^+^]: 80
Morgan *et al.*^40^	Cycling (indoor) and fluid restriction	Baseline (78.7)	—	—	—	BS_osm_°: 287 BS[Na^+^]°: 140	—	—	—
		Post 60 min	—	—	—	BS_osm_°: 292 BS[Na^+^]°: 141.5	5 1.5	1.7 1.1	—
		Post 120 min	—	—	—	BS_osm_°: 295 BS[Na^+^]°: 142	8 2	2.8 1.4	SW_osm_: 172 SW[Na^+^]: 91.1
*Walsh et al.*^39^	Cycling (indoor) and fluid restriction	BMc: 1.8% Post 60 min	UR[Na^+^]: 9.9	—	—	—	—	—	SW[Na^+^]: 108.9

Abbreviations: Abs (absolute difference to baseline); BM_C_ (body mass change, percentage change); BP_osm_/BS_osm_ (blood plasma/serum osmolality, mOsmol/kg); BP[Na^+^]/BS[Na^+^] (blood plasma/serum sodium concentration, mmol/l); Rel (relative difference to baseline); SW_osm_ (sweat osmolality, mOsmol/kg), SW[Na^+^] (sweat sodium concentration, mmol/l); UR_osm_ (urine osmolality, mOsmol/kg); UR[Na^+^] (urine sodium concentration, mmol/l); UR_sg_ (urine-specific gravity, g/ml); values estimated out of figures° italic=athletes/soldiers.

**Table 3:** General characteristics of the included studies—tear

*Study*	*Dehydration procedure*	*Monitoring time (body mass)*	*Urine*	*Abs*	*Rel*	*Blood*	*Abs*	*Rel*	*Tear*	*Abs*	*Rel*
Fortes *et al.*^31^	Cycling (indoor)	Baseline (68.1)	UR_sg_°: 1.006	—	—	BP_osm_: 288	—	—	TE_osm_: 293	—	—
	and fluid restriction	BMc 1%	UR_sg_°: 1.008	0.002	0.2	BP_osm_°: 289	1	0.3	TE_osm_°: 299	6	2
		BMc 2%	UR_sg_°: 1.017	0.011	1.1	BP_osm_: 292	3	1.4	TE_osm_°: 300	7	2.4
		BMc 3%	UR_sg_: 1.021	0.015	1.5	BP_osm_: 297	9	3.1	TE_osm_°: 305	12	4.1
		BMc (overnight) 3.5%	UR_sg_: 1.026	0.02	2	BP_osm_: 297	9	3.1	TE_osm_: 304	11	3.8
*Ungaro et al.*^27^	Cycling (indoor) and fluid restriction	Baseline (75.7)	UR_sg_°: 1.006	—	—	BP_osm_°: 292	—	—	TE_osm_°: 296	—	—
		BMc 1%	UR_sg_: 1.012	0.006	0.6	BP_osm_: 293	1	0.3	TE_osm_: 299	3	1
		BMc 2%	UR_sg_: 1.020	0.014	1.4	BP_osm_: 295	3	1	TE_osm_: 301	5	1.7
		BMc 3%	UR_sg_: 1.021	0.015	1.5	BP_osm_: 297	5	1.7	TE_osm_: 302	6	2

Abbreviations: Abs (absolute difference to baseline); BM_C_ (body mass change, percentage change); BP_osm_ (blood plasma osmolality, mOsmol/kg); Rel (relative difference to baseline); TE_osm_ (tear osmolarity, mOsmol/l, euhydrated: <310 mOsmol/l); UR_sg_ (urine-specific gravity, g/ml); values estimated out of figures° italic=athletes.
